# Research protocol - Evaluating data quality in the Netherlands Perinatal Registry (Perined): A data comparison study using hospital records from the IUGR Risk Selection (IRIS) study

**DOI:** 10.12688/f1000research.150160.2

**Published:** 2025-01-09

**Authors:** Hilde Plomp, Corine Verhoeven, Lilian Peters, Aimée van Dijk, Wes Onland, Ank de Jonge, Jens Henrichs

**Affiliations:** 1 Midwifery Academy Amsterdam Groningen, Inholland University of Applied Sciences, Amsterdam, The Netherlands; 2 Midwifery Science, Amsterdam University Medical Centres, Amsterdam, The Netherlands; 3 Department of Obstetrics and Gynecology, Maxima Medical Centre, Veldhoven, The Netherlands; 4 Division of Midwifery, School of Health Sciences, University of Nottingham, Nottingham, England, UK; 5 Department Primary and Long-term Care, University Meidcal Center Groningen, Groningen, The Netherlands; 6 Amsterdam Public Health, Amsterdam, The Netherlands; 7 Perined, Utrecht, The Netherlands; 8 Emma Children's Hospital, Amsterdam University Medical Centres, Amsterdam, The Netherlands; 9 Reproduction and Development, Amsterdam University Medical Centres, Amsterdam, The Netherlands

**Keywords:** Perined, perinatal database, data quality, completeness, reliability, agreement

## Abstract

**Background:**

The quality of registry based studies depends largely on the data accuracy of the registries. The Dutch Perinatal Registry (Perined) is a nationwide database comprising perinatal data digitally provided by different healthcare providers. Perined data are used for comparing outcomes across regions and healthcare institutions as well as for quality analyses and research purposes. However, little is known about the data quality of the Perined database. Therefore, this research protocol depicts our proposed study assessing the quality of Perined data compared to hospital records and case report forms (CRFs) that were part of the IUGR Risk Selection (IRIS) study.

**Methods:**

In the planned comparison study data from Perined and the IRIS Study will be used. The IRIS study was a large cluster-randomized trial investigating the effectiveness of routine third trimester ultrasonography in reducing severe adverse perinatal outcomes among Dutch low-risk pregnant women. A subsample of the IRIS study of neonates being at risk of severe adverse perinatal outcomes and their mothers will be used. Baseline demographic data were collected by midwives from participating women at inclusion (around 22 weeks’ gestation) using CRFs, and in-depth neonatal and maternal clinical data were retrieved from hospital records by trained research assistants. These latter IRIS study data were linked and compared to Perined data. Completeness of Perined data will be calculated for every variable. The reliability will be assessed as the percent of agreement between Perined and hospital record data or the CRF-based data. Additionally, intra-class correlation coefficients will be calculated for continuous variables, and Kappa and ‘Prevalence-and-Bias-Adjusted Kappa’ will be calculated for categorical variables.

**Discussion:**

The results of the planned comparison study will provide users of Perined data insight in its data quality. This will serve as an example of the accuracy of registry based data used in maternal and neonatal care research.

## Introduction

Many countries have a nationwide perinatal database containing data on maternal and neonatal outcomes, such as mode of birth and birth weight. ^1^^–^^3^ Countries use national perinatal data for monitoring outcomes, quality analyses and research. The data can also be used for comparing results between hospitals, midwifery practices and regions within the country and between countries. ^2^^,^^4^^–^^6^ For example, EURO-PERISTAT merges aggregated data of all EU members states, Norway, Iceland, Switzerland and the United Kingdom for comparing maternal and neonatal outcomes between the countries. ^2^ For these purposes, high quality of the data included in these national databases is essential. Nevertheless, there is a lack of research and insufficient insight into the data quality of these databases.

In 2018, a study was published about the completeness of birth registries worldwide. Phillips et al. concluded that in 2011 only 40% of the total births in the world were registered in a public database, although registration was better in high income countries. ^1^ In 2019, two Canadian studies were published on the data accuracy of one of two of the Ontario Birth Registries: the BORN (Better Outcomes Registry and Network. ^7^^,^^8^ Miao et al. compared the BORN birth registry with the general Canadian clinical hospital database (n = 404,439) by evaluating data on key maternal and neonatal outcomes. ^7^ Dunn et al. compared data from BORN with data extracted from hospital records (n = 927), and focussed on key outcomes, e.g. labour type and gestational age at birth, but also considered more specific maternal and neonatal outcomes, e.g. ‘pain relief measures during newborn screening’ or ‘serum bilirubin’. ^8^ Both studies found an overall good agreement for most key maternal and neonatal outcomes, e.g. almost perfect agreement for gestational age at birth and date of birth. ^7^^,^^8^ Remarkably, in the study of Miao et al. the lowest Kappa was found for stillbirth or live birth (Kappa 0.74), because of discrepancies in the coding of stillbirths between the data sources. ^7^ The study by Dunn et al. using hospital records as comparison found lower agreement for more specific outcomes, e.g. intention to breastfeed and maternal smoking, than for key outcomes. ^8^ The BORN birth registry has also been compared to another Canadian birth registry: CIHI-DAD (Canadian Institute for Health Information Discharge Abstract Database). ^9^ When lower agreement between variables was found, disagreement could often be explained by differences in coding or differences in definitions of the compared variables. ^9^ Another study conducted in Finland in 2002 on the Finnish medical birth registry showed that check-box questions improved data quality, compared to open-ended questions. ^10^ In addition to research on the accuracy of birth registry databases, studies concerning the data quality of non-perinatal databases showed that the overall completeness, validity, and reliability of national medical databases in high income countries was good and has improved over the years. ^11^^–^^15^

The planned study focuses on the evaluation of the quality of data obtained from the Netherlands Perinatal Registry (Perined). ^16^ Such an evaluation is crucial as Perined serves important healthcare-related and scientific purposes. The aim of Perined is to improve the quality of Dutch perinatal care. ^16^ The database is used for comparing outcomes between regions, midwifery practices and hospitals, for quality analyses to improve perinatal care and research. ^4^ The database contains data on pregnancy, birth and neonatal outcomes. ^4^ The data are collected by midwives, obstetricians and paediatricians as part of their regular practice, and a selection of items is shared with Perined. ^16^ In 2020 97% of all Dutch births were registered in Perined. ^4^

Several large-scale Dutch studies used Perined data, for example the DELIVER (Data EersteLIjns VERloskunde) study, ABCD (Amsterdam Born Children and their Development) study and the IRIS (IUGR RIsk Selection) study. ^17^^–^^19^ These studies all had more than 5000 participants. Data in these studies were collected in different ways and Perined data were used for key maternal and neonatal outcomes. Other studies are completely based on Perined data, e.g. the development of the latest Dutch birthweight charts. ^20^

This illustrates that Perined data are regularly used for research purposes. However, to the best of our knowledge, the data quality of the Perined database has never been assessed in a study. Therefore, the aim of the planned study is to assess the data quality of the Perined database, by assessing the completeness and agreement of the data as compared to perinatal and maternal peripartum variables based on the original hospital records and baseline demographic data from the IUGR Risk Selection (IRIS) study.

## Methods

### Design

This research protocol depicts a data comparison study concerning a subsample of data based on the IRIS study. ^19^^,^^21^ To assess agreement, data from Perined will be compared with data extracted from hospital records from the participating mothers and/or neonates or with demographic baseline data collected via Case Report Forms (CRFs) at enrolment of participating women (around 22 weeks’ gestation) completed by the midwives together with the participants. ^19^^,^^21^ The data extracted from the hospital records and CRF-based demographics of the IRIS study are used as reference standards. In short, the IRIS study was a multi-centre nationwide stepped wedge cluster randomized trial investigating the effectiveness of routine ultrasound screening in the third trimester of pregnancy in reducing severe adverse perinatal outcomes in low risk pregnancies. ^19^ Routine third trimester ultrasound screening was compared with standard care. Sixty primary care midwifery practices in the Netherlands and a total of 13,520 women with a low risk singleton pregnancy participated in the IRIS study. The women were enrolled between February 2015 and February 2016. Data of 13,024 (96.3% of 13,520) participating mothers and neonates were linked to Perined data. ^19^ Data extracted from the hospital records (n=2884) were collected for neonates at risk for severe adverse perinatal outcomes and for mothers with an at risk neonate and/or with indications of peripartum pathology or hospitalization (see section *data collection and selection* for more details and variable operationalization).

### Perined data

**Figure 1.**
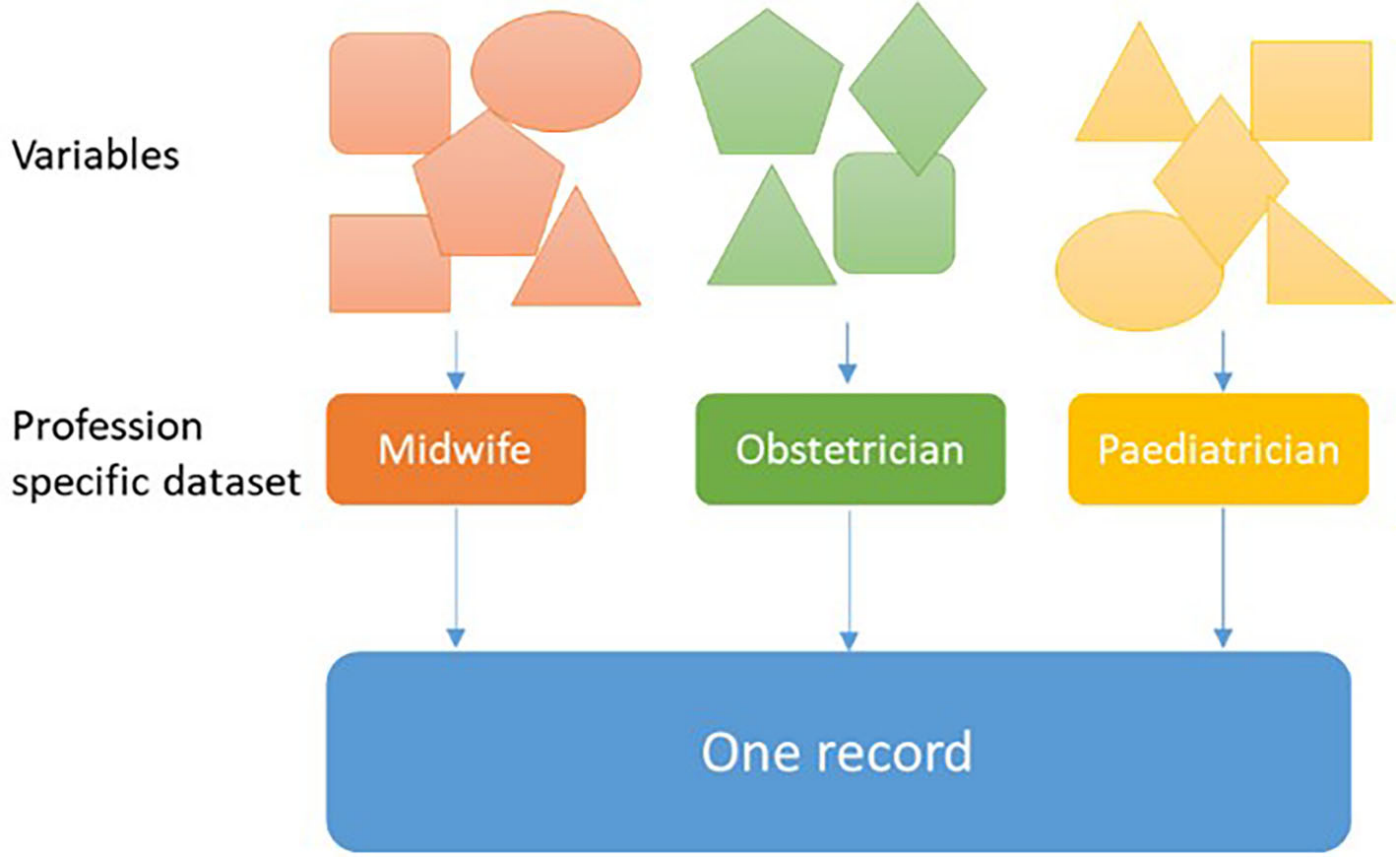
Perined links datasets and creates one record for all neonates and their mothers. ^24^

Data for this study were collected in 2015 and 2016. In 2016 98% of the Dutch births were registered in Perined. ^22^ Each profession registers a set of variables. ^23^ Perined links the datasets of each profession and creates one record for all neonates and their mothers ( Figure 1). ^24^ Some variables are uniquely registered by the respective healthcare providers of one profession, whereas others (e.g. type of birth) are registered by healthcare providers of multiple professions. For every variable that is registered by more than one profession, Perined uses a decision tree to determine which value is leading in the Perined record. ^24^ The records used in this study are all records with information from more than one healthcare profession, thus having a complex data structure. In 2015 and 2016 Perined started with a new registration approach for hospitals in the Netherlands. Some hospitals used the new approach, while others still used the old approach. The old and the new approach differ in the variables that are recorded, some variables have been added in the new approach (e.g. length and weight of the pregnant woman), while others have been removed (e.g. method of conception). Also, for some variables answer options have been changed. For the IRIS study, most of the data were provided according to the old approach. The data provided according to the new approach have been recoded by Perined according to the specifications of the old approach.

### Data collection and selection

**Figure 2.**
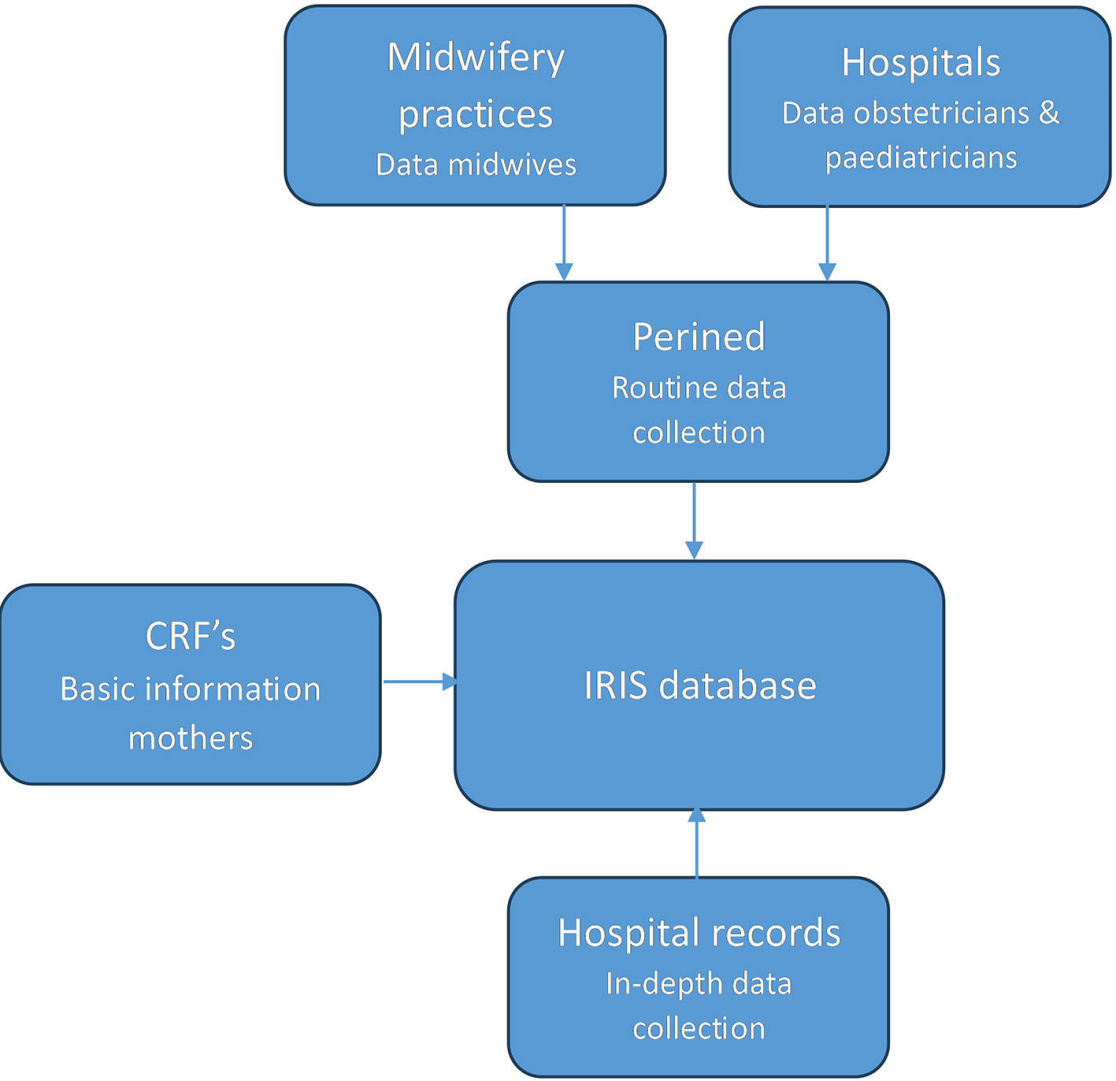
Data sources of the planned study.

Perined data and extracted hospital record data for the planned comparison study are selected using a subsample of the IRIS study. Figure 2 shows the used data sources for this study. For the IRIS study hospital records were selected for in-depth data collection based on certain criteria for the Perined or survey data. If the Perined record suggested a case of perinatal death (between 28 weeks pregnancy and 7 days postpartum), a low Apgar score (<4) at five minutes, a birth weight between percentile 2.3 and 5 and neonatal hospitalization for more than three days, or a birth weight < percentile 2.3, data from hospital records were extracted. Hospital records were also selected based on a longitudinal survey; a subsample of women in the IRIS study (n= 1949) took part in this survey. ^21^ The survey data were used to identify cases at risk for a severe adverse perinatal outcome. When mothers indicated in the survey that they had a consultation or referral of care to obstetrician-led care, or that their baby visited a paediatrician or neonatologist, they were included for the in-depth data collection from hospital records if they met certain criteria. Maternal indications to be included in the in-depth data collection included maternal hospitalization during pregnancy and/or postpartum or maternal hospitalization for more than 48 hours after birth without medical intervention or an hospitalization of more than 72 hours related to a caesarean section. ^25^ Trained research assistants performed the data extraction. After data extraction from the hospital records, the amount of error in data entry was analysed. Double entry analyses were performed on hospital records of 111 women. The incidence of data entry error was 3.2% overall, 3.7% for maternal data and 2.6% for neonatal data. ^19^

Extracted data from the selected hospital records and CRFs were to those from the Perined records of the same mothers and neonates in order to compare these two types of records. Data managers linked the records by matching several demographic variables (e.g. date of birth of the mother and postal code). Variables will be included in the current study if they are available in both Perined and either the extracted hospital record data or the CRFs. Table 1 and Table 2 show which variables will be compared. The categories of the variables will be created with data driven approaches. We expect that the data for these variables as derived from the respective source can be coded into the categories as shown in Table 1 and Table 2, or are already available in this way. The syntax comprising the respective coding steps will be published, after publication of the final results as planned in the current study. In case a good comparison between certain variables is not possible, they will be removed and not include in the comparative analyses.

**Table 1.** Example|Descriptive statistics of the variables to be studied based on Perined and the hospital records/CRFs.

Variable ^1^					
Maternal variables (n=)	Perined data source ^2^	Perined n (%)	Missing n	Hospital records or CRFs* n (%)	Missing n
Maternal age *(mean (SD))*					
Maternal ethnicity					
Dutch					
Other					
Parity					
0					
1					
2					
3					
4					
>4					
Responsible profession at start of labour					
Midwife-led care					
Obstetric-led care					
Referral of care from midwife-led care to obstetric-led care during labour					
Yes					
No					
Responsible profession at birth					
Midwife-led care					
Obstetric-led care					
Start of labour					
Spontaneous					
Induction of labour					
Planned caesarean section					
Type of birth					
Spontaneous					
Assisted vaginal birth					
Planned caesarean section					
Unplanned caesarean section					
Epidural/spinal analgesia					
Yes					
No					
Augmentation of labour					
Yes					
No					
Postpartum haemorrhage (>1000 cc blood loss)					
Yes					
No					
Episiotomy					
Yes					
No					
Third or fourth degree perinatal trauma					
Yes					
No					

Neonatal variables (n=)	Perined data source ^1^	Perined n (%)	Missing N	Hospital records n (%)	Missing n
Gestational age at delivery (days) *(median (IQR))* ^b^					
Sex					
Boy					
Girl					
Inconclusive					
Birth weight (grams) *(mean (SD))*					
Apgar score 5 minute postpartum <7					
Yes					
No					
If pH-value is determined, value *(mean (SD))*					
Consultation by the paediatrician and/or hospitalization of the neonate					
Yes					
No					
Perinatal death					
No					
Antenatal death					
Natal death					
Neonatal death (up to 7 days postpartum)					

^1^ Final choices for the categorization per variable will be made when doing the analysis. An attempt will be made to stay as close as possible to the Perined categories as used in the Perined data.

^2^ As discussed in the methods section Perined variables can be registered by one healthcare profession, but also by more than one. This column will show the data source(s) of the variable.

**Table 2.** Example|Degree of completeness and the agreement between Perined and hospital records/CRFs to be studied.

Variable					
Maternal and birth variables	Degree of completeness Perined (%)	Percent agreement (%)	Kappa (95% CI) or ICC* (95% CI)	PABAK	Cases (n)
Maternal age					
Maternal ethnicity					
Parity					
Responsible profession at start of labour					
Referral of care from midwife-led care to obstetric-led care during labour					
Responsible profession at birth					
Start of labour					
Type of birth					
Epidural/spinal analgesia					
Augmentation of labour					
Postpartum haemorrhage (>1000 cc blood loss)					
Episiotomy					
Third or fourth degree perinatal trauma					
***Neonatal variables***					
Gestational age at birth (in days)					
Sex					
Birth weight (in grams)					
Apgar score 5 minutes postpartum <7					
pH-value umbilical cord determined					
If pH-value is determined. value					
Consultation by the paediatrician and/or hospitalization of the neonate					
Perinatal death					
No					
Antenatal death					
Natal death					
Neonatal death (up to 7 days postpartum)					

Records with missing data for some of the variables will be included in the planned study, to assess the degree of completeness of the different variables in Perined.

### Statistical analyses

The data in the IRIS study are not always coded in the same way as those in Perined. Recoding of the variables will be necessary to make comparisons possible, this will be done in Rstudio 4.2.1 and IBM SPSS statistics 28. We will stay as close as possible to the Perined categories. If too much recoding is required to compare the variables, no comparison will be considered. All statistical analyses will be performed in Rstudio 4.2.1. PABAK will be calculated in Rstudio 4.2.1 with the formula 2*((a+d)/(a+b+c+d))-1, based on a 2x2 table. ^26^ For ordinal scales, PABAK will be calculated using the PABAK-OS calculator. ^27^

By using descriptive statistics, frequencies and percentages will be presented for all categorical variables. For continuous variables (e.g. birthweight, Apgar score) with a normal distribution, means and standard deviations (SD) will be calculated. Medians and interquartile ranges (IQR) will be reported for continuous variables not normally distributed.

The completeness of the selected variables in Perined will be calculated as the number of patients with information recorded in Perined divided by the total number of patients in the hospital records dataset or CRF based dataset.

The agreement of the categorical variables will be assessed as the percent agreement and Cohen’s Kappa with 95% confidence interval (CI). Cohen’s Kappa is a statistical measure for dichotomous, categorical and nominal variables to examine the proportion of agreement corrected for the proportion of agreement that could be expected by chance. ^8^^,^^28^ The following criteria to assess the strength of agreement will be used: Kappa coefficient ≤ 0 poor, 0.01-0.20 slight, 0.21-0.40 fair, 0.41-0.60 moderate, 0.61-0.8 substantial, 0.81-0.99 almost perfect. ^29^^,^^30^ The advantage of using Kappa as a measure of agreement is the correction for chance. The disadvantage of using Kappa is the extreme sensitivity for unequal distributions. When one of the values is very common and the other very rare, Kappa is drastically lowered, even when the agreement is high, this is defined as the Kappa’s paradox. ^31^ Therefore, the ‘Prevalence-Adjusted-Bias-Adjusted-Kappa’ (PABAK) will also be calculated for all categorical variables as this measure can account for unequal distributions of the test variables. ^26^ For PABAK, the same strength of agreement criteria as for KAPPA will be used.

For continuous variables, the agreement between Perined and the extracted hospital record data will be assessed using the percent agreement and intra-class correlation coefficient (ICC) with 95% CI. The ICC is a correlation coefficient which tests whether there is agreement between two continuous variables measuring the same outcomes in two different data sources. The ICC is an appropriate statistical measure of reliability for continuous data. ^32^ ICC values range between 0 (no agreement) and 1 (total agreement). The following criteria to assess the level of agreement will be used: < 0.5 poor, 0.5-0.75 moderate, 0.75-0.9 good, > 0.90 excellent. ^32^

## Conclusion and discussion

The planned study with data of the IRIS study provide a unique opportunity to validate the Perined dataset on a large scale. In recent years the Dutch Perined database has been increasingly used for various purposes, e.g. benchmarking between Dutch regions, benchmarking between nations, and various types of research. However, so far, little is known about the data quality of Perined despite its importance for healthcare evaluation and research purposes.

The planned study will have some strengths and limitations. The greatest strength of this study is that using a large-scale nationally representative low risk pregnancy population in the Netherlands provides the rare opportunity to validate the quality of Perined data. A potential limitation is that the data collection for the IRIS study was conducted in the years 2015 and 2016. These years were so-called ‘transition years’ for the Dutch perinatal registration during which Perined implemented a new dataset system. For the IRIS study the ‘old’ way of data registration and coding were used. Data from participating care institutions that already used the new approach were recoded to fit in the old dataset format as depicted above. A priori one might assume that this may have influenced the degree of missingness in some perinatal variables. The linkage rate between Perined and the complete IRIS study data was very high (96.3%) as compared to a recent study with a much lower linkage rate (78.5%) using the ‘new data registration approach’ only. ^19^^,^^33^ Data used for this study were mostly based on complex records reported by different healthcare professionals, see Figure 1. In several instances, information on a certain variable, e.g. birth weight, was entered by several healthcare professionals so that a decision rule was used by Perined determining from which healthcare profession data should be used for a specific variable. One might assume that the accuracy of variables registered by only one healthcare profession may be more accurate than for those variables entered by multiple professions. However, this is unavoidable as for a large group of women and their offspring in ante- and postnatal care referred from midwifery-led care to obstetrician-led care data is generally entered into the Perined database by multiple healthcare professions. ^4^

By investigating the data quality of Perined using data from the IRIS study the planned comparison study can inform researchers about the reliability and usefulness of the Perined-based neonatal and maternal variables addressed in this study. This information will help researchers to make adequate choices for Perined derived outcome variables to be used in future studies. Next to this the results of the current study will help medical software developers to improve data-extraction procedures, and Perined to improve data-collection procedures. In addition, the results of this study can illustrate how national birth registries can be improved to enhance quality improvement activities in perinatal care research.

### Ethical considerations

This study is a secondary analysis of the IRIS study which was approved by the Dutch Institutional Review Board of the VU Medical University Centre Amsterdam on the 17 ^th^ of December 2013 (reference number 2013.409). This study was performed in line with the ethical principles established by the World Medical Association in the Declaration of Helsinki of Ethical Principles for Medical Research Involving Human Subjects. ^34^ Women gave permission to link their records, the linking was performed by data managers of the IRIS study with the support of Perined. After linking the data all identifiable patient information about patients was removed. Analyses for the planned study will be done with a completely anonymous data set, without any identifying variables (e.g. patient names, date of births and study ID).

### Consent to participate

Between February 2015 and February 2016, pregnant women in participating midwifery practices who fulfilled the inclusion criteria were informed about the study and received an information leaflet from their midwife during the first consultation. All participants gave written informed consent for data usage.

## Author contributions

**Plomp H**: Methodology, Writing – Original Draft Preparation, Writing – Review & Editing; **Verhoeven CJM, de Jonge A, Henrichs J:** Conceptualization, Supervision, Writing – Original Draft Preparation, Writing – Review & Editing **. Peters L, van Dijk AE, Onland W:** Writing – Review & Editing

## Data Availability

The existing ethical approval for the IRIS study does not permit publication of individual participant level data. Requests for a de-identified dataset require a Data Transfer Agreement that is in line with the European Union’s General Data Protection Regulation (GDPR) and can be sent to the corresponding author ( j.henrichs@amsterdamumc.nl).

## Software availability

All software code that will be used for this study based on IBM SPSS statistics 28 and Rstudio 4.2.1 can also be made available by the coordinator of the IRIS study.

Rstudio 4.2.1 is open access. ^35^ IBM SPSS statistics 28 is proprietary software, PSPP is a non-proprietary alternative. ^36^^,^^37^

National perinatal registration data from the Netherlands is available publicly from Perined at www.peristat.nl.
