# Supplementary material for: Research protocol - Evaluating data quality in the Netherlands Perinatal Registry (Perined): A data comparison study using hospital records from the IUGR Risk Selection (IRIS) study
Source: F1000Res. 2025 Jan 9;13:686. Originally published 2024 Jun 24. [Version 2] doi: 10.12688/f1000research.150160.2 (PMC11822247; doi:10.12688/f1000research.150160.2)
Supplement: Peer review history

# Reviewer report: Reviewer response for version 2

**DOI:** 10.5256/f1000research.175292.r357737

**Published:** 2025-02-12

**Authors:** Elizabeth Kathleen Darling

**Affiliations:** 1 McMaster University, Hamilton, Ontario, Canada

Thank you for your thoughtful and detailed responses to my previous comments. You have addressed all of my suggestions in a satisfactory manner.

Is the study design appropriate for the research question?

Yes

Is the rationale for, and objectives of, the study clearly described?

Yes

Are sufficient details of the methods provided to allow replication by others?

No

Are the datasets clearly presented in a useable and accessible format?

Not applicable

Reviewer Expertise:

NA

I confirm that I have read this submission and believe that I have an appropriate level of expertise to confirm that it is of an acceptable scientific standard.

# Reviewer report: Reviewer response for version 2

**DOI:** 10.5256/f1000research.175292.r357738

**Published:** 2025-02-03

**Authors:** Patricia Lee King

**Affiliations:** 1 Medical Social Sciences, Northwestern University - Chicago (Ringgold ID: 205058), Chicago, Illinois, USA; 2 University of Chicago Pritzker School of Medicine, Chicago, USA

Thank you to the authors for the edits based on reviewer feedback. They were responsive to the requests. My only remaining comment is that I do still think the use of the word study in the title and the paper impacts clarity that this is a protocol for a study that will happen and confuses the reader on what is a plan versus what is completed. For example, with the following edits to the title I'm more clear on what is a compelted study and what is work forthcoming:

 A research protocol to evaluate data quality in the Netherlands Perinatal Registry (Perined): A protocol for a data comparison study using hospital records from the IUGR Risk Selection (IRIS) study

Is the study design appropriate for the research question?

Partly

Is the rationale for, and objectives of, the study clearly described?

Partly

Are sufficient details of the methods provided to allow replication by others?

Yes

Are the datasets clearly presented in a useable and accessible format?

Not applicable

Reviewer Expertise:

quality improvement, perinatal health

I confirm that I have read this submission and believe that I have an appropriate level of expertise to confirm that it is of an acceptable scientific standard.

# Reviewer report: Reviewer response for version 1

**DOI:** 10.5256/f1000research.164702.r295929

**Published:** 2024-07-16

**Authors:** Elizabeth Kathleen Darling

**Affiliations:** 1 McMaster University, Hamilton, Ontario, Canada

This manuscript presents a study protocol for a data quality study that will compare data collected in the Netherlands Perinatal Registry (Perined) with data collected via hospital records and case report forms for participants in a large cluster-randomized trial (the IRIS study). The authors make a strong case for the importance of high-quality national perinatal registry data to support quality improvement and research, and they successfully argue that the use of high-quality data collected as part of an RCT presents a unique opportunity to assess the quality of Perined data.

 The objectives of the study are clearly stated and the study design is appropriate to address the objectives. The data sources and the methods originally used to collect the data are adequately described. The plans for statistical analysis are appropriate and are well described. The dummy tables that are provided (Tables 1 & 2) are appropriate to present the planned analyses. (Revising the titles of these tables might help to make their purpose more clear to the reader.)

 The protocol could be strengthened by the addition of some key information. First, no details are provided regarding how the data sources will be linked. The authors cite a prior study that linked Perined and IRIS data, implicitly demonstrating feasibility, but do not explain what variable(s) will be used for linkage. Furthermore, under 'Ethical considerations', the authors state that the analyses will be done using an anonymous data source, without patient names and study ID, which again raises the question of how the data will be linked. If a previously linked data set is being used, this should be explained. Second, the protocol for the study should include the algorithms that will be used to recode some variables to allow for comparison between two sources. This could be presented as the first table (or a supplementary table if it is very lengthy), and should ideally include the name of the variable in each source and the rules that will be applied to to recode the comparator variables to align them with Perined variables when applicable. This level of detail is necessary to permit replication of the study. Finally, it would be helpful for the reader if you could add another sentence or two to explain the difference between the old and new registration approaches for hospitals that are/were used for Perined. This additional contextual detail would help the reader understand the potential implications of the change in approach.

 Given the citation of two similar studies in the introduction, the authors may be interested in this recent publication that also examines data accuracy of the Ontario Birth Registry (BORN) -Ref [1]

 I look forward to reading the findings from this study once it is completed.

Is the study design appropriate for the research question?

Yes

Is the rationale for, and objectives of, the study clearly described?

Yes

Are sufficient details of the methods provided to allow replication by others?

No

Are the datasets clearly presented in a useable and accessible format?

Not applicable

Reviewer Expertise:

midwifery services, large database research, access to care, health equity

I confirm that I have read this submission and believe that I have an appropriate level of expertise to confirm that it is of an acceptable scientific standard, however I have significant reservations, as outlined above.

## References

[1] : Defining a low-risk birth cohort: a cohort study comparing two perinatal data sets in Ontario, Canada. *Int J Popul Data Sci* .2024;9(1) : 10.23889/ijpds.v9i1.2364 2364 10.23889/ijpds.v9i1.2364 38505395 PMC10949111

# Response

**Published:** 2024-11-25

**Authors:** Hilde Plomp

**Affiliations:** Midwifery Science, Amsterdam UMC, Amsterdam, The Netherlands

Dear Elizabeth K. Darling,

 We would like to thank you for reading and reviewing the article, for the positive evaluation and compliments concerning our article, and for the additional thoughtful comments and suggestions. Below is a point-by-point reply to your comments. We indicated the start of your comments by using your initials (EKD) and our respective reply starts with HP (i.e. Hilde Plomp the first author answering on behalf of all the authors). Changed text passages, which are presented below, are indicated by using quotation marks, and for the convenience of the reviewer, we also refer to the respective pages and lines in the manuscript where these passages can be found.

**Reviewer:** Revising the titles of these tables might help to make their purpose more clear to the reader.

**Author Response:**  We have changed the titles of the tables to make it more clear to the reader that these are example tables and what they contain. For example, the title of Table 1 now reads:

*“* *Table 1. Example|Descriptive statistics of the variables to be studied based on  Perined and the hospital records/CRFs.* *”*

**Reviewer: **First, no details are provided regarding how the data sources will be linked. The authors cite a prior study that linked Perined and IRIS data, implicitly demonstrating feasibility, but do not explain what variable(s) will be used for linkage.”

**Author Response:** We have added some additional information about the data linkage procedure in the Methods section, as shown below (see also page 6 of the manuscript):

*“Extracted data from the selected hospital records and CRFs were linked to those from the Perined records of the same mothers and neonates in order to compare these two types of records. Data managers linked the records by matching several demographic variables (e.g. date of birth of the mother and postal code). Variables chosen for analysis will be included in the planned study if they are available in both Perined and either the extracted hospital record data or the CRFs.”*

**Reviewer:** Furthermore, under 'Ethical considerations', the authors state that the analyses will be done using an anonymous data source, without patient names and study ID, which again raises the question of how the data will be linked. If a previously linked data set is being used, this should be explained.

**Author Response:** We have added some extra information in the ‘Ethical considerations’ section. It is important to note that the datasets were not completely anonymous to the data managers involved in our study. However, researchers receive anonymized datasets for data-analysis purposes. Our additions to the ‘Ethical considerations’ section are shown below (see also page 14):

*“Women gave permission to link their records, the linking was performed by data managers of the IRIS study with the support of Perined. After linking the data all identifiable patient information about patients was removed. Analyses for the planned study will be done with a completely anonymous data set, without any identifying variables (e.g.  patient names, date of births and study ID).”*

**Reviewer:** Second, the protocol for the study should include the algorithms that will be used to recode some variables to allow for comparison between two sources. This could be presented as the first table (or a supplementary table if it is very lengthy), and should ideally include the name of the variable in each source and the rules that will be applied to recode the comparator variables to align them with Perined variables when applicable. This level of detail is necessary to permit replication of the study.

**Author Response: **We cannot include the requested syntax in the current article as the stage of our planned work does not allow this. The syntax of the recoding will be published as a supplementary file included in the subsequent scientific article reporting our final results. Until we have completed the variable coding and data-analysis, we cannot guarantee full precision regarding the best recoding method for each variable *(see also page 6):*

*“The categories of the variables will be created with data driven approaches. We expect that the data for these variables as derived from the respective source  can be coded into the categories as shown in Tables 1 and 2, or are already available in this way.  The syntax comprising the respective coding steps will be published, after publication of the final results as planned in the current study. In case a good comparison between certain variables is not possible, they will be removed and not included in the comparative analyses.”*

**Reviewer:** Finally, it would be helpful for the reader if you could add another sentence or two to explain the difference between the old and new registration approaches for hospitals that are/were used for Perined. This additional contextual detail would help the reader understand the potential implications of the change in approach.

**Author Response:** We have added some additional information on this aspect in the Methods section (see also page 5):

*“In 2015 and 2016 Perined started with a new registration approach for hospitals in the Netherlands. Some hospitals used the new approach, while others still used the old approach. The old and the new approach differ in the variables that are recorded, some variables have been added in the new approach, while others have been removed. Also, for some  variables  answer options have been changed. For the IRIS study, most of the data were provided according to the old approach. The data provided according to the new approach have been recoded by Perined according to the specifications of the old approach.”*

**Reviewer:** Given the citation of two similar studies in the introduction, the authors may be interested in this recent publication that also examines data accuracy of the Ontario Birth Registry (BORN) -Ref [1].

**Author Response: **thank you for the suggestion. We have added your recent publication in the Introduction (see also page 4).

*“The BORN birth registry has also been compared to another Canadian birth registry: CIHI-DAD (Canadian Institute for Health Information Discharge Abstract Database) [Darlings et al., 2024]. When lower agreement between variables was found, disagreement could often be explained by differences in coding or differences in definitions of the compared variables [bron: Darlings, 2024].”*

 We hope that our changes are satisfactory. We are looking forward to your evaluation.

 Kind regards, on behalf of all authors,

 Hilde Plomp

# Reviewer report: Reviewer response for version 1

**DOI:** 10.5256/f1000research.164702.r295927

**Published:** 2024-07-13

**Authors:** Patricia Lee King

**Affiliations:** 1 Medical Social Sciences, Northwestern University - Chicago (Ringgold ID: 205058), Chicago, Illinois, USA; 2 University of Chicago Pritzker School of Medicine, Chicago, USA

This article describes a research protocol for a planned evaluation of data quality of the Netherlands Perinatal Registry (Perined) comparing it to the IRIS study database. Intra-class correlation and Kappa will be calculated to determine accuracy. This research protocol will assess the accuracy of Perined which is widely used for research and public health decision making by comparing it to clinical data. 

 This is a detailed description of a study protocol of great importance to public health and research 9- the clinical accuracy of a large data set used for national decision making. It could be strengthened by clarifying in the article that this is a planned study/ study protocol being described. The structure of the article leaves the reader looking for results until they reach the conclusion section. The reference to "study protocol" article category at the title is probably not enough for clarity. The Tables, instead of example, could be labeled data collection forms/templates for further clarity.

Is the study design appropriate for the research question?

Partly

Is the rationale for, and objectives of, the study clearly described?

Partly

Are sufficient details of the methods provided to allow replication by others?

Yes

Are the datasets clearly presented in a useable and accessible format?

Not applicable

Reviewer Expertise:

quality improvement, perinatal health

I confirm that I have read this submission and believe that I have an appropriate level of expertise to confirm that it is of an acceptable scientific standard, however I have significant reservations, as outlined above.

# Response

**Published:** 2024-11-25

**Authors:** Hilde Plomp

**Affiliations:** Midwifery Science, Amsterdam UMC, Amsterdam, The Netherlands

Dear Patricia Lee King,

 We would like to thank you for the positive evaluation and compliments concerning our article and for your thoughtful comments.  We agree with your suggestion to better clarify that the current paper concerns a study protocol, this was made clear in the title, but possibly insufficiently in the rest of the article. Therefore, we now explicitly state in several relevant places in the text and in the tables that the current paper concerns a study protocol (see the manuscript). Moreover, we have made some additional changes to the text by for example by using the term “planned study” to indicate that the current work only concerns a study protocol.

 We hope that our changes are  satisfactory. We look forward to your evaluation.

 Kind regards, on behalf of all authors,

 Hilde Plomp
